# 高效液相色谱-大气压化学电离-串联质谱法同时测定动物源食品中复硝酚钠的3种组分

**DOI:** 10.3724/SP.J.1123.2022.03006

**Published:** 2022-12-08

**Authors:** You ZOU, Linzhi SHAO, Cao LAN, Simin CHEN

**Affiliations:** 广州海关技术中心食品与化妆品检测研究所, 广东 广州 510623; Food and Cosmetics Testing Institute, Guangzhou Customs Technology Center, Guangzhou 510623, China

**Keywords:** 高效液相色谱-串联质谱, 大气压化学电离, 复硝酚钠, 动物源食品, high performance liquid chromatography-tandem mass spectrometry (HPLC-MS/MS), atmospheric pressure chemical ionization (APCI), sodium nitrophenolate, foodstuffs of animal origin

## Abstract

复硝酚钠(SNP)是一种生长调节剂,在我国动物源食品检测中被列为禁用药物。由于复硝酚钠痕量分析方法不成熟,至今尚无标准检测方法,因此建立复硝酚钠中3种组分(5-硝基愈创木酚钠、对硝基酚钠和邻硝基酚钠)同时检测的方法对我国动物源食品中复硝酚钠残留水平的控制、检测标准的制定和政府相应管理措施的实行采取具有一定的理论和现实意义并兼具创新性。研究建立了高效液相色谱-大气压化学电离-串联质谱(HPLC-APCI-MS/MS)对猪肉、鸡肉、鱼肉和肝脏中复硝酚钠3种组分残留量的检测方法。样品采用氢氧化钠溶液提取,用盐酸调节pH值为酸性后,加氯化钠使溶液饱和,再用乙腈溶液反萃两次后,合并上清液并加入饱和氯化钠溶液,再经正己烷液液萃取除脂后吸出中间乙腈层,浓缩并定容后,以甲醇-水溶液作为流动相进行梯度洗脱,采用CORTECT C_18_色谱柱分离,大气压化学电离,在多反应监测(MRM)负离子模式下测定,外标法定量分析。5-硝基愈创木酚钠、对硝基酚钠和邻硝基酚钠分别在0.5~10、1.0~20和2.5~50 μg/L范围内线性良好,定量限分别为1.0、2.0和5.0 μg/kg,在定量限、2倍定量限和10倍定量限加标水平上的回收率分别为81.5%~98.4%、81.5%~102%和81.4%~95.1%,相对标准偏差分别为1.51%~5.98%、1.10%~8.85%和0.91%~8.61%(*n*=6),均符合要求,能够满足动物源食品中复硝酚钠残留量的检测要求。

## Abstract (en)

Sodium nitrophenolate (SNP) is a widely used universal growth regulator consisting of 5-nitroguaiacol sodium (5NG), 4-nitrophenol sodium (PNP), and 2-nitrophenol sodium (ONP). SNP has a positive influence on plants and animals as a feed additive that accelerates growth but is potentially hazardous to humans. SNP has been reported to be cytotoxic and mutagenic, which may increase the risk of cancer and pose a great threat to food safety. There are neither mature detection nor standard methods for the trace analysis of SNP in animal food. Therefore, the development of an accurate and precise analytical method is imperative. This innovative method has theoretical and practical significance for the control of SNP residues, offering advantages such as cost-effectiveness and time efficiency. It will be beneficial for the establishment of detection standards and management measures in foodstuffs of animal origin.

In this study, a reliable method for the simultaneous determination of SNP residues in animal food (porcine muscle, chicken tissue, fish, and liver) was developed. For realizing the perfect limit of quantification, the application of back extraction coupled with high performance liquid chromatography-atmospheric pressure chemical ionization-tandem mass spectrometry (HPLC-APCI-MS/MS) was proposed to combine high sensitivity and high selectivity. The optimal method was as follows. First, 2.0 g samples were extracted with 10 mL 0.5 mol/L sodium hydroxide solution, followed by adjustment of the pH to acidity with 3 mol/L hydrochloric acid and the addition of sodium chloride (5.0 g) to saturate the inorganic phase. After back-extraction twice with 16 mL acetonitrile, the solution was merged and again saturated with 5 mL of sodium chloride solution. Second, the merged organic phase was cleaned up with 10 mL of *n*-hexane for defatting. The middle acetonitrile layer was then concentrated to nearly 1.5 mL at 40 ℃ in a N_2_ stream before dilution with the mobile phase to a volume of 3.0 mL and filtered. Finally, the analytes were separated on a C_18_ column (100 mm×4.6 mm, 3 μm) and subjected to gradient elution with a mixed solution of methanol and water. Mass spectrometric analysis, which was quantified using the external standard method, was carried out with an atmospheric pressure chemical ionization negative ion source and based on multiple reaction monitoring (MRM) mode. The key parameters, such as the extraction solvent, extraction steps, and purification method, were optimized.

The calibration curves were linear in the ranges of 0.5-10 (5NG), 1.0-20 (PNP), and 2.5-50 μg/L (ONP) with correlation coefficients greater than 0.9999. The limit of quantification (LOQ) for 5NG was 1.0 μg/kg, double for PNP, and five times for ONP. The recoveries of the three different concentration levels in all the four matrices were in the range of 81.5%-98.4%, 81.5%-102%, and 81.4%-95.1%. The repeatability, expressed as the relative standard deviations (RSDs) of the three compounds, ranged from 1.51% to 5.98%, 1.10% to 8.85% and 0.91% to 8.61% (*n*=6). The developed method is characterized by an excellent purification effect, sensitivity, and accuracy. This method is suitable for the simultaneous and quantitative determination of SNP residues in foodstuffs of animal origin.

复合硝基酚钠也称复硝酚钠,是几种含硝基苯酚钠盐(有的产品是铵盐)的复合型植物生长调节剂,最早于20世纪90年代由日本旭化学工业株式会社研发,商品名为“爱多收”“爱丰收”和“Atonik”,由5-硝基愈创木酚钠(5NG)、对硝基酚钠(PNP)和邻硝基酚钠(ONP)按照一定比例构成^[1]^。因具有高效、低毒、廉价等优点,复硝酚钠在农业以及养殖业领域适用非常广泛,既可单独使用,也可作为增效剂与农药、肥料、饲料等配合使用。与饲料复配使用时,复硝酚钠能促进动物的食欲及其对营养的吸收,加快生长发育,同时增强动物的免疫能力,提高肉、蛋、奶、皮、毛的产量和质量^[2⇓-4]^。近年来有研究表明,植物生长调节剂在农作物中的残留通过食物链进入人体会造成一定程度的危害。复硝酚钠虽然毒性较低,但长期食用含复硝酚钠残留的动植物食品仍对人体健康存在潜在危害。美国环保局(EPA)已将邻硝基酚和对硝基酚列为“优先控制污染物”^[5]^,对硝基酚也被列入中国优先控制污染物黑名单^[6]^。欧盟委员会发布(EU)2021/590号条例,修改制定了复硝酚钠在某些产品中的最大残留限量^[7]^,在作为商品销售的猪、牛、羊、马、家禽等可食用组织中复硝酚钠的最大残留限量(以5-硝基愈创木酚钠、对硝基酚钠和邻硝基酚钠之和计,并以5-硝基愈创木酚钠表示)均为0.03 mg/kg。2019年底,我国农村农业部250号公告也正式将复硝酚钠列入食用动物禁止使用的药品及其他化合物清单^[8]^。

目前,国内尚未有农畜产品中复硝酚钠残留监控的检测标准。查阅已发表的文献,复硝酚钠在动物源食品中的检测方法大多为气相色谱法和高效液相色谱法。其中,气相色谱法需对样品进行酸化或衍生化后才能测定^[9]^,前处理复杂,准确度较低,报道的定量限较高,均为10 μg/kg;高效液相色谱法是检测复硝酚钠比较常用的方法^[10,11]^,但现有文献报道的方法普遍存在样品分析时间长、灵敏度较低等问题,且仅报道了检出限,不能满足定量检测的要求。相比而言,液相色谱-串联质谱法在检测动物样品中的药物残留特别是在禁用药物的痕量分析中具有更好的灵敏性和更高的特异性^[12⇓⇓⇓⇓-17]^,但却几乎无人用于复硝酚钠中3种组分的同时检测。原因在于ONP的分子结构中,硝基基团的氧原子与邻位酚羟基的氢原子形成分子内氢键,使酚羟基的氢原子不易被电离,导致了ONP的极性极弱,在电喷雾电离(ESI)模式下的灵敏度比其余两个组分低了3个数量级,从而影响整个方法的灵敏度^[18]^。然而,大气压化学电离(APCI)与电喷雾电离的原理不同,主要用于弱极性和小分子化合物的分析。Xing等^[19]^采用了带APCI源的高效液相色谱-串联质谱对水产品中的复硝酚钠进行痕量分析,灵敏度很高。

本研究采用碱性溶液提取样品,去除大部分水溶性杂质,后续通过调节pH值、乙腈反萃、正己烷脱脂净化,能有效避免样品提取过程中杂质的干扰,减少基质效应(ME),再以APCI为电离方式,建立了高效液相色谱-大气压化学电离-串联质谱法同时对复硝酚钠3种组分进行测定,以提高方法的正确度和灵敏度,为制定标准提供了较为可靠的参考依据,填补了动物源食品中复硝酚钠残留量测定的技术空白,具有广阔的应用前景。

## 1 实验部分

### 1.1 仪器、试剂与材料

QTRAP 5500三重四极杆质谱仪,带大气压化学电离源,Analyst仪器控制及Multiquant数据处理软件(美国SCIEX公司); UFLC-XR液相色谱仪(包括输液泵LC-20AD、自动进样器SIL-20AC、柱温箱CTO-20AC)(日本岛津公司); BT223S型电子天平(德国Sartorius公司); 3-30K高速离心机(美国Sigma-Aldrich公司); Biotage Turbo Vap氮吹仪(南京新飞达光电科学技术有限公司); MS 3 basic涡旋混合器(德国IKA公司)。

5-硝基愈创木酚钠(CAS号:67233-85-6,纯度:96.15%)、对硝基苯酚钠(CAS号:824-78-2,纯度:80.47%)(德国Ehrenstorfer公司);邻硝基苯酚钠(CAS号:824-39-5,纯度:99.9%,北京曼哈格生物科技有限公司);乙腈、甲醇(HPLC级,美国Fisher Scientific公司);盐酸(优级纯,广州化学试剂厂);实验室用水为一级水(符合GB/T 6682-2008一级水要求);其他未作特殊说明的试剂均为分析纯。

### 1.2 样品的前处理

#### 1.2.1 提取

称取样品2.00 g(精确至±0.02 g)于50 mL离心管中,加入0.5 mol/L氢氧化钠溶液10 mL,在涡旋混合器上混匀30 s后,振荡提取15 min,以3 mol/L的盐酸溶液调整pH至3.0后加入5.0 g氯化钠,涡旋1 min,再加入8 mL乙腈,振荡提取20 min,以4000 r/min离心5 min。取上清液于另一50 mL离心管中,残渣继续加入8 mL乙腈,振荡提取20 min后以4000 r/min离心5 min,合并上清液,备用。

#### 1.2.2 净化

在备用液中先加入5 mL饱和氯化钠溶液,再加入10 mL正己烷,振荡后以4000 r/min离心5 min,取中间乙腈层于15 mL离心管中,于40 ℃下氮吹至约1.5 mL后加一级水定容至3 mL,以0.2 μm滤膜过滤,供液相色谱-串联质谱测定。

### 1.3 分析条件

#### 1.3.1 色谱条件

CORTECT C_18_色谱柱(100 mm×4.6 mm, 3 μm);流动相A:水,流动相B:甲醇;流速:0.80 mL/min;柱温:40 ℃;进样量:20 μL。梯度洗脱程序:0~2.0 min, 10%B; 2.0~3.0 min, 10%B~55%B; 3.0~6.5 min, 55%B; 6.5~9.5 min, 55%B~95%B; 9.5~9.6 min, 95%B~10%B, 9.6~13.0 min, 10%B。

#### 1.3.2 质谱条件

离子源:大气压化学电离源,负离子扫描模式;喷雾电压(IS): -4500 V;离子源温度(TEM): 500 ℃;气帘气(氮气)压力:40 kPa;碰撞气(氮气)压力:中等;雾化气(氮气)压力:50 kPa;辅助加热器压力(氮气): 60 kPa;放电电流:-2 μA;多反应监测(MRM)模式。

优化后的3种目标化合物的保留时间与质谱参数见表1。

**表1:** MRM模式下5NG、PNP和ONP的保留时间与质谱参数

Compound	t_R_/min	Parent ion (m/z)	Product ions (m/z)	DP/V	CEs/eV
5-Nitroguaiacol sodium (5NG)	4.89	168.0	153.0^*^, 123.0	-55	-18, -27
4-Nitrophenol sodium (PNP)	4.96	138.0	108.0^*^, 92.0	-90	-20, -23
2-Nitrophenol sodium (ONP)	5.82	138.0	108.0^*^, 92.0	-90	-22, -30

* Quantitative ion; DP: declustering potential; CE: collision energy.

## 2 结果与讨论

### 2.1 提取溶剂的选择

复硝酚钠易溶于水,可溶于乙醇、甲醇、丙酮等有机溶剂。研究首先选取常用的有机试剂如甲醇、乙腈、0.1%乙酸乙腈和乙酸乙酯作为提取溶剂进行提取效率对比试验,直接加入等量有机试剂提取,再浓缩上机,考察4种有机试剂对3种化合物回收率的影响,结果见图1。采用乙酸乙酯提取的溶液不能直接用于质谱分析,需要氮吹至干转换溶剂,但通过标准品验证,吹干复溶操作会导致目标化合物损失严重,特别是邻硝基酚钠损失超过50%。其余3种有机提取试剂中,0.1%乙酸乙腈的提取效率最高,接近95%;乙腈次之,能达到90%。但直接加入有机溶剂容易使蛋白质和脂肪含量较高的肌肉组织结团,难以分散,需要通过高速匀浆才能使其均匀分散,增加了前处理的复杂性。而且,用乙腈和0.1%乙酸乙腈直接提取虽然效率高,但提取浓缩后基质效应很大,需要设计更多的净化步骤去除基质效应,增加了实验的不确定性。复合硝酚钠中的3种组分在碱性条件下均以负离子形式存在,亲水性很强,因此尝试采取先用氢氧化钠溶液进行提取,再用有机溶剂反萃取的两步提取方法进行试验。强碱不仅能够溶解大部分的水溶性杂质,而且能够使蛋白质变性,形成可以通过离心即能除去的沉淀,而微溶于水中的脂肪颗粒则可以通过后续用正己烷进一步除去。被提取出来的溶液通过盐酸调节成酸性,此时,复硝酚钠的3种组分呈现分子形态^[20]^,疏水性强,易富集于有机溶剂。考虑到要在质谱仪上进行负离子检测,且提取液吹干会造成损失,故选择提取效率较高、能用于质谱分析的乙腈作为萃取剂进行反萃,再通过加入氯化钠使溶液饱和,进一步降低目标化合物在水相中的残留,使其最大化地在有机相中富集,提高萃取效率,减少基质效应。故本研究采用碱溶液作为提取溶剂再用乙腈反萃取替代直接加入有机溶剂的提取方式。

**图1:**
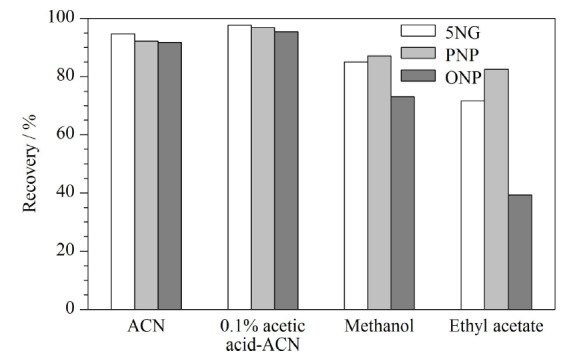
不同提取溶剂对5NG、PNP和ONP回收率的影响

### 2.2 净化方法的选择

提取液的净化是样品前处理过程中关键的一步,直接关系到方法的定量限和回收率。动物基质成分复杂,强碱溶液作为提取溶剂能把部分水溶性杂质、蛋白质和脂肪去除。乙腈作为反萃溶剂能把微溶于水相的脂肪颗粒提取出来,故要设计净化方式除去溶于乙腈中的少量脂肪。研究选取正己烷和几种不同品牌的除脂柱(Oasis PRiME HLB小柱和EMR-Lipid小柱)进行脱脂试验。结果显示,正己烷的液液分配净化方式即可达到脱脂效果;而使用除脂柱前,要求备用液含20%水才能使脂肪被有效吸附,但此操作却不利于后续氮吹浓缩和定容。因此,净化步骤采用简单快捷的正己烷液液分配方法去除脂溶性杂质。

### 2.3 流动相的优化

#### 2.3.1 流动相选择

根据复硝酚钠的性质,比较了甲醇、乙腈和不同浓度的甲酸铵和水溶液作为流动相时的分离效果。结果表明,当水相使用不同浓度的甲酸铵溶液时,邻硝基酚钠和对硝基酚钠均未能有效分离;当水相使用一级水时,3种组分有效分离且灵敏度达到最高。当有机相选择使用乙腈时,出现了基线不平的现象,且邻硝基酚钠出峰前有干扰峰无法分离。通过比较保留时间、响应值、背景干扰和洗脱能力,流动相体系最终确定为甲醇-水,不但节省了溶剂配制的时间,且峰形好,灵敏度高。

#### 2.3.2 洗脱方式的优化

在等度洗脱实验中,当甲醇大于55%时,复硝酚钠的3种组分便能有效分离,单纯对标准溶液进行色谱分离,重复性好,未见异常。但在实际样品的色谱分离中发现:甲醇比例达到55%~80%时,前一个样品在色谱柱上分离后因等度洗脱的有机相比例不高,洗脱能力较弱,导致极性较大的杂质仍被保留在色谱柱上,干扰了下一个样品的色谱分离。然而,当甲醇比例大于80%时,复硝酚钠3种组分在色谱柱上均不保留,出峰时间小于2 min,容易被共流出物干扰影响定量。因此,优化了梯度洗脱方式进行样品的色谱分离,确保当次进样的目标分析物和杂质均能被洗脱,不会对下一个样品分离造成影响,且保留时间适中,能最大限度地避免杂质干扰。

### 2.4 离子源的选择

研究对比了液相色谱-串联质谱法常用的电喷雾电离和大气压化学电离两种电离模式。其中,5-硝基愈创木酚钠和对硝基酚钠在ESI模式下响应很高,但基质效应也很强。而邻硝基酚钠则因其硝基上的氧原子与邻位酚羟基上的氢原子形成了分子内氢键,产生邻位效应^[21]^,酚羟基上的氢原子电离效率差,导致灵敏度比对硝基酚钠低了至少3个数量级,在样品检测中难以适应禁用兽药的痕量检测要求,故容易被忽略和遗漏。APCI由于电离原理与ESI不同,离子转化率更高,主要适用于弱极性化合物的检测^[22]^。复硝酚钠3种组分在APCI模式下分离的色谱峰见图2,可见响应良好,均能达到痕量分析水平,且不易形成多电荷分子碎片,离子竞争少,杂峰较少,基质效应也较弱,故选择使用APCI模式电离。

**图2:**
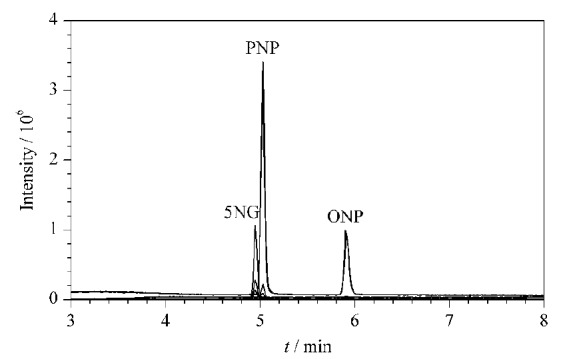
APCI模式下5NG、PNP和ONP混合标准溶液的总离子流色谱图

### 2.5 基质效应评价

动物源食品的样品基质复杂,进行质谱分析时容易受到基质中共流出物的干扰,影响方法的灵敏度和重复性,故需要对基质效应进行评价。根据实际情况及可操作性,选择按Matuszewski等^[23]^提出的提取后添加法评价基质效应。以阴性空白基质提取液作为溶剂,配制5.0 μg/L的混合标准溶液1,测定其峰面积为*A*;以定容液为溶剂配制5.0 μg/L的混合标准溶液2,测定其峰面积为*B*。ME=*B/A*×100%,结果见图3。其中,ME>100%为基质增强效应,ME<100%为基质抑制效应,ME=100%可以当作基质效应不存在,这是最为理想的一种情况,但实际操作中,一般ME值在85%~115%之间,则认为基质效应不明显^[24]^。

**图3:**
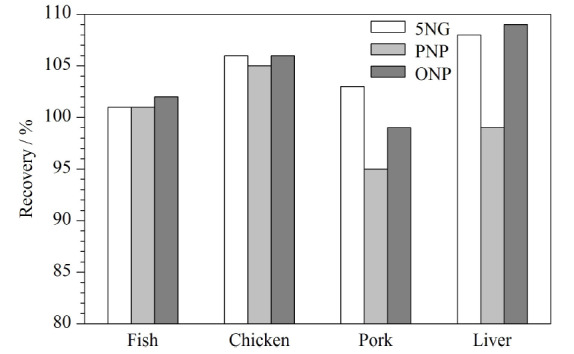
在不同基质中5NG、PNP和ONP的基质效应

针对复硝酚钠这类弱极性化合物,本实验采用了相比ESI源离子化效率更高、基质效应较小的APCI源^[25⇓-27]^,由图3可见,3种目标化合物在4种基质中的基质效应在95%~109%以内,表明优化前处理方法后提取所得样液对目标化合物的基质影响不大,因此无需使用基质匹配标准曲线校正,提高了方法在日常多样化样品中的检测效率。

### 2.6 线性范围与定量限

在设定的实验条件下,配制一系列不同浓度的标准溶液,以仪器响应峰面积对复硝基酚钠3种组分的质量浓度进行线性回归。结果表明,5-硝基愈创木酚钠、对硝基酚钠和邻硝基酚钠分别在0.5~10、1.0~20和2.5~50 μg/L范围内的线性关系良好,相关系数均大于0.9999。当样品中的复硝酚钠含量超过其线性范围时,可适当加大样品的稀释倍数,使其上机浓度落在线性范围内。在阴性空白样品中从低到高添加不同水平的混合标准工作溶液,以定量离子对色谱峰的信噪比(*S/N*)大于10确定方法的定量限,结果见表2。

**表2:** 5NG、PNP和ONP的回归方程、线性范围、相关系数和定量限

Compound	Regression equation	Linear range/(μg/L)	r^2^	LOQ/(μg/kg)
5NG	Y=1.90×10^5^X-6.27×10^3^	0.5-10	0.99999	1.0
PNP	Y=2.32×10^5^X+1.30×10^5^	1.0-20	0.99998	2.0
ONP	Y=4.54×10^4^X+3.77×10^3^	2.5-50	0.99991	5.0

Y: peak area; X: mass concentration, μg/L.

以鱼肉为代表基质,添加定量限水平浓度的标准溶液时,3种目标化合物的定量离子对MRM色谱图见图4。

**图4:**
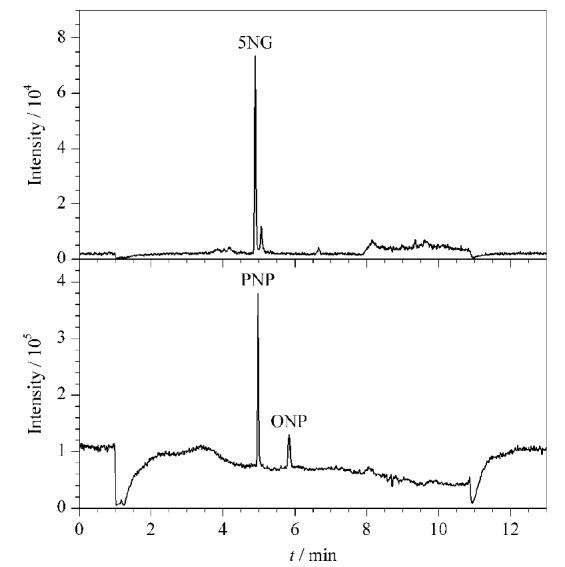
空白鱼肉添加定量限水平5NG、PNP和ONP时的提取离子色谱图

### 2.7 方法的准确度

采用空白样品加标方法进行加标回收和精密度试验。分别对猪肉、鸡肉、鱼肉和肝脏进行3个水平(定量限、2倍定量限和10倍定量限)加标,每个水平平行测定6次,计算平均回收率和相对标准偏差。结果表明,复硝酚钠的3种组分在4种基质中的平均回收率在81.4%~102%之间,相对标准偏差在0.91%~8.85%之间(见表3),符合GB/T 27404-2008《实验室质量控制规范 食品理化检测》^[28]^规定的要求,可用于动物源食品中复硝酚钠的定量检测。

**表3:** 猪肉、鸡肉、鱼肉、肝脏样品中5NG、PNP和ONP的加标回收率及相对标准偏差(n=6)

Compound	Added/ (μg/kg)	Pork		Fish		Chicken		Liver	
		Recovery/%	RSD/%	Recovery/%	RSD/%	Recovery/%	RSD/%	Recovery/%	RSD/%
5NG	1.0	95.0	5.98	88.6	3.20	85.4	2.74	84.6	2.70
	2.0	92.0	5.30	82.8	1.51	81.6	1.75	83.0	2.10
	10.0	98.4	3.79	82.8	1.87	81.5	1.87	84.1	4.06
PNP	2.0	100	5.17	88.0	8.85	83.3	1.11	92.3	1.82
	4.0	101	2.77	84.3	2.34	87.1	3.81	84.5	2.65
	20.0	102	1.70	81.5	1.86	86.9	2.62	85.6	2.83
ONP	5.0	95.1	8.61	90.6	1.95	82.8	0.91	87.3	5.90
	10.0	84.8	5.88	83.3	2.77	84.9	1.78	83.7	2.93
	50.0	85.5	4.85	82.1	1.82	83.6	2.63	81.4	1.39

### 2.8 实际样品检测

用本方法对市售猪肉、鸡肉、鱼肉和肝脏样品共96份进行测定。结果显示,其中1个鸡肉样品检出对硝基酚钠,含量为27.0 μg/kg, 5-硝基愈创木酚钠和邻硝基酚钠均未检出。其余样品也均未检出3种复硝酚钠。

## 3 结论

本文结合动物源性基质的特点和复硝酚钠的理化性质,对前处理方法和仪器方法进行了优化,建立了HPLC-APCI-MS/MS法,用于同时测定动物源食品中复硝酚钠的3种组分。该方法检测成本低,实验周期短,在多种基质中的加标回收率高,重复性好,定量科学准确,基质效应不明显,能够为动物源食品中复硝酚钠的残留检测提供可靠的技术支持。
